# Some Scientific Aspects of Insanity

**Published:** 1892-02-20

**Authors:** 


### Some Scientific Aspects of Insanity.

### XIII.—DEMENTIA.

Dementia means mental weakness. There are two great forms of dementia. In the first, which is called primary dementia, the mind is weak, because the brain, the physical basis of mind, has been imperfectly developed. In the second, which is known as secondary dementia, the mind is weak because the minute structure of its physical basis the brain has in many parts been destroyed in consequence of, or secondarily to, some destructive agency, such as an acute attack of insanity. Primary dementia, then, includes the two great classes of hereditary mental weakness, viz., Idiots and Imbeciles.

I. Idiots.—An idiot may be defined as a person whose mental powers have not developed beyond those usually met with in infancy or early childhood. It must not, however be supposed that an idiot always remains a child. For although, as a whole, the nervous development is arrested, it is also irregularly developed, so that parts of the body and mind are more developed than other parts. The bodily stature is, as a rule, low and the bodily configuration misshapen. The human instincts—those higher instincts which distinguish man from the beast —are apt to be wanting altogether, or, at best, to be only rudimentarily present. The tastes of an idiot are usually depraved. The faculty of reasoning which man shares to a great extent with the higher divisions of the lower animals is here, unfortunately, much impaired; and thus, while an idiot by position in the human family occupies a high position, he is, on account of his want of reasoning ability, rendered lower than some of the brutes. This lack of reasoning power, or, in other words, mental weakness, unfits the idiot to take any position in the society of his fellows, and what is equally important it unfits him to gain a livelihood of any description.

II. Imbeciles.—The distinction between idiots and imbeciles is not a sharp one, the one class runs into the other insensibly, but the order of mental development of an imbecile is higher than that of an idiot. An imbecile may be defined as an individual whose mental development has been arrested in childhood, and though, as in the case of idiotcy, the mental condition of an imbecile cannot be compared to that of a child at any particular age on account of the unequal and irregular development of the mind, yet there is always present the mental weakness or mental deficiency which indicates that the person has never attained to the full and average enjoyment of mental power. There are different degrees of imbecility, extending from those who are little better than idiots, up to those about whom externally there is nothing particular to remark, but who are regarded by their friends as having "a want." The great distinction between idiots and imbeciles is that the latter are generally able, however imperfectly, to occupy some position in society, and to earn a livelihood, however small or inadequate it may be.

While many imbeciles of the higher order never require medical attention or nursing, all idiots and the great majority of imbeciles require to be placed under the care of others. Where nature has not provided the material to work upon the cure of this large division of the insane is manifestly impossible. Very much, however, can be done to ameliorate and improve their condition. Unlike those patients who suffer from secondary dementia, those who have the misfortune to labour under primary dementia are usually endowed with a considerable degree of curiosity, a certain amount of interest in their surroundings, and a strongly imitative disposition. With the aid of these three positive qualities much good can be done by establishing in the individual habits of cleanliness, tidiness, a sense of order and self-restraint. Many very hopeless-looking cases can in this way be got to do a little work, and many can be trained to the extent of being able to appreciate and enjoy suitable amusements. In accordance with the degree of mental capacity present in each case must the thoughtful attendant attempt to extend the education of the patient. There is no better proof of the influence of attention and the constant exercise of supervision upon the habits of these cases than the rapid and marked deterioration which takes place when from any cause the exercise of care is relaxed or abandoned.

The subcutaneous injection of arsenic in preference to its administration by the mouth as less poisonous seems to have many advocates in America. When introduced into the skin, arsenic enters the general circulation without taking a sojourn in the liver and upper intestines, so producing a directly beneficial effect. Every solution, however, does not appear suitable for subcutaneous injection.

